# Hold the door! Stomatal defense also protects against mites

**DOI:** 10.1093/plphys/kiae212

**Published:** 2024-04-10

**Authors:** Manuel González-Fuente

**Affiliations:** Assistant Features Editor, Plant Physiology, American Society of Plant Biologists; Faculty of Biology & Biotechnology, Ruhr-University Bochum, Bochum D-44780, Germany

With the exception of a few passionate acarologists, mites are probably not among anyone's favorite bugs. They colonize our skin (causing diseases), our beds (causing allergies), our pets (feeding on their blood), and even cute little honeybees (contributing to the colony collapse disorder responsible for tremendous bee losses worldwide). Nobody has asked (on the record) plants about their opinion on mites, but if someone would have, they would probably share the same lack of fondness toward these little bugs, as mites also parasitize plants. In fact, phytophagous (i.e. plant-feeding) mites pose serious threats to many plant species, including many economically important crops. One of the most ubiquitous and problematic phytophagous mites is the two-spotted spider mite, *Tetranychus urticae*, that infects more than 1500 different plant species (Migeon and Dorkeld 2024). *T. urticae* pierces and feeds on individual leaf parenchyma cells, causing chlorosis and cell death. Its fully sequenced genome, the first from an arachnid, together with its ability to feed on *Arabidopsis thaliana* have made *T. urticae* a model species for the study of plant–herbivore interactions (Grbić et al. 2011). Nevertheless, phytophagous mite infectious strategies and plant defense mechanisms against them are much less studied and characterized than those of more canonical pathogens such as bacteria or fungi.

In this issue of *Plant Physiology*, Rosa-Díaz et al. (2024) revealed the defensive role of abscisic acid (ABA)-mediated stomatal closure for protection against *T. urticae* infection (Fig.). They observed that, upon mite infestation, plants closed their stomata. Simultaneously, mite infestation also triggered an accumulation of defense-related hormones such as jasmonic, salicylic, and abscisic acids. Using high-resolution FRET-based sensors, the authors observed that ABA accumulated upon infection in all leaf tissues but more strongly in stomatal cells, coherent with the role of this hormone in stomatal closure upon stress (Sawinski et al. 2013).

**Figure.**
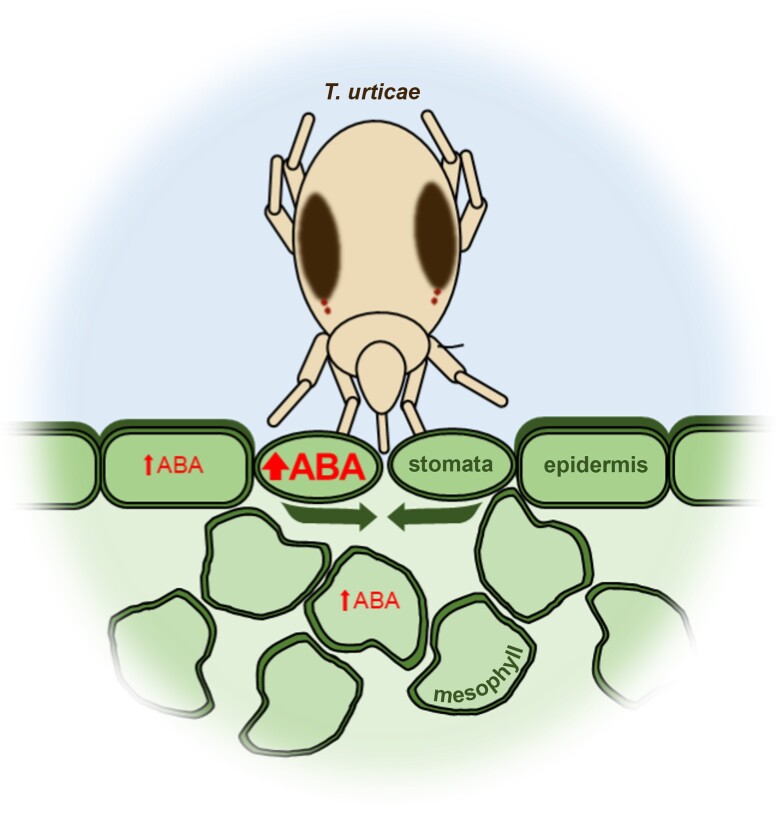
*Tetranychus urticae* triggers ABA-mediated stomatal closure. *T. urticae* induces the local accumulation of ABA in all leaf tissues but more strongly in stomata cells, inducing their closure as a protective measure to prevent *T. urticae* proliferation.

To discern whether ABA-mediated stomatal closure upon infection is a virulence strategy of the mite or a defense reaction from the plant, ABA synthesis-defective and over-accumulator mutants were infested. The ABA synthesis mutant was more damaged and harbored higher deposition of *T. urticae* eggs, whereas the ABA over-accumulator was more tolerant. This was further corroborated with exogenous application of ABA, which not only maintained the stomata closed but also limited the damages caused by the mites. Accordingly, chemical opening of stomata increased the mite-derived damages. Moreover, plants with lower and higher stomatal density were more tolerant and susceptible to mite infestation, respectively. Altogether, these results favor the hypothesis that ABA-mediated stomatal closure is indeed a plant defensive mechanism against *T. urticae*.

Prevention is better than cure: preinvasive defenses are a crucial first step to prevent pathogen proliferation (Cerutti et al. 2017). As the entry point of many different pathogens, stomatal immunity has been more classically studied in the context of avoiding the pathogen colonization (Sawinski et al. 2013). As such, stomatal aperture/closure has been a continuous arms race between plants and pathogens (Melotto et al. 2006; Ye et al. 2020; Raffeiner et al. 2022). However, more recently it has been proposed that stomata also play a role in later stages of infection (Roussin-Léveillée et al. 2022). But what happens when the pathogen is substantially larger than a stomata? Do stomata also play a role in the defenses against such pathogens? Previous reports showed that stomata also respond to insects and that, in turn, insects can manipulate stomata (Lin et al. 2022). On this matter, the work by Rosa-Díaz et al. (2024) adds to this, proving that ABA-mediated stomatal closure is also an effective plant defensive mechanism against mites. This opens new lines of research, as the mite signal(s) recognized by the plant to trigger this defense mechanism, as well as the possible mite countersignal(s) to prevent it, are still unknown. Also, it needs to be assessed whether this defensive mechanism is specifically effective against *T. urticae* or in general against mites. This would set the groundwork to include stomatal aperture and density as parameters to consider during resistance breeding against devastating phytophagous mites.
